# Potential deleterious effects of paracetamol dose regime used in Nigeria versus that of the United States of America

**DOI:** 10.1016/j.toxrep.2022.04.025

**Published:** 2022-04-27

**Authors:** Samuel James Offor, Cecilia Nwadiuto Amadi, Ifeyinwa Chijioke-Nwauche, Jose E. Manautou, Orish E. Orisakwe

**Affiliations:** a Department of Pharmacology and Toxicology, Faculty of Pharmacy, University of Uyo, Uyo, Akwa Ibom State, Nigeria; b Department of Experimental Pharmacology & Toxicology, Faculty of Pharmacy, University of Port-Harcourt, Port-Harcourt, Rivers State, Nigeria; c Department of Clinical Pharmacy, Faculty of Pharmacy, University of Port Harcourt, Port Harcourt 5323, Rivers State, Nigeria; d Department of Pharmaceutical Sciences, University of Connecticut, Storrs, CT 06269, USA; e African Centre of Excellence for Public Health and Toxicological Research (ACE-PUTOR), University of Port Harcourt, PMB, 5323 Port Harcourt, Choba, Nigeria

**Keywords:** Paracetamol, Hepatotoxicity, Acetaminophen, Nigeria, Liver

## Abstract

Paracetamol, also known as acetaminophen (N-acetyl-para-aminophenol, APAP) is the world’s most used over-the-counter analgesic-antipyretic drug. Despite its good safety profile, acetaminophen can cause severe hepatotoxicity in overdose, and poisoning from paracetamol has become a major public health concern. Paracetamol is now the major cause of acute liver failure in the United States and Europe. This systematic review aims at examining the likelihood of paracetamol use in Nigeria causing more liver toxicity vis-à-vis the reduced maximum recommended daily adult dose of 3 g for the 500 mg tablet. Online searches were conducted in the databases of PubMed, Google Scholar and MEDLINE for publications using terms like “paracetamol toxicity,” “acetaminophen and liver toxicity,” “paracetamol and liver diseases in Nigeria,” and other variants. Further search of related references in PubMed was carried out, and synthesis of all studies included in this review finalized. There were 94 studies that met the inclusion criteria. Evaluation of hepatic disorder was predicated mostly on a constellation of clinical features and limited clinical laboratory investigations. Determination of blood paracetamol concentration was rarely reported, thus excluding paracetamol poisoning as one of the likely causes of liver disorders in Nigeria. In Nigeria and elsewhere, several factors are known to increase paracetamol’s predisposition to liver injury. They include: the over-the-counter status of paracetamol, use of fixed-dose combinations of paracetamol with other drugs, malnutrition, dose miscalculations, and chronic alcohol consumption. The tendency to exceed the new paracetamol maximum daily dose of 3 g in Nigeria may increase its risk for hepatotoxicity than observed in the United States of America known for emphasizing lower dose of the drug. In addition to recommending the new maximal daily paracetamol dose allowance, the historical maximum daily adult dose of 4 g should be de-emphasized in Nigeria.

## Highlights

• Paracetamol can cause severe hepatotoxicity in overdose.
• Paracetamol is the major cause of acute liver failure in the United States and Europe.
• Determination of blood paracetamol concentration is rarely reported in Nigeria.
• Paracetamol poisoning as one of the likely causes of liver disorders in Nigeria.
• Paracetamol maximum daily dose of 3 g in Nigeria may increase hepatotoxicity.

## Graphical Abstract

**Figure:**
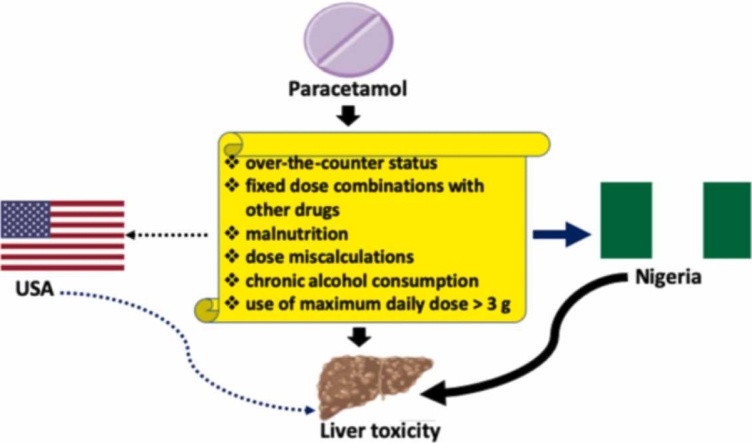

## 1 Introduction

### 1.1 Paracetamol: therapeutic uses and hepatotoxic potentials

Paracetamol, also known as acetaminophen (N-acetyl-para-aminophenol, APAP), is the most widely used over-the-counter analgesic-antipyretic drug worldwide, and the most commonly used medication in paediatrics [1], [2], [3], [4]. Paracetamol was used in the United States of America to replace the drug, phenacetin, which was no longer in use because of its toxicity to the kidney [3]. It is a non-opioid analgesic useful in the treatment of mild/moderate pains like headache, postpartum pain, myalgia, and in many conditions where treatment with aspirin is not effective [5]. Several over-the-counter and prescription preparations are made solely of paracetamol or its combination with other drugs [3], and many users are unaware of its content in these combination medications [6], [7], [8]. This is particularly problematic with users that buy multiple over-the counter multi-symptoms medicines unaware that they contain paracetamol and without consulting their pharmacist or physician. Paracetamol became the preferred drug for treating fever and pains in paediatric populations because of the occurrence of Reye's syndrome with paediatric use of aspirin [9]. In children less than 6 years, paracetamol is the most common drug involved in cases of overdose [10], [11]. When ingested in large quantities, paracetamol can cause serious hepatotoxicity despite showing relatively good safety profile at therapeutic levels [12]. Although the risk of paracetamol toxicity is commonly seen in children, poisoning episodes are often more serious and fatal in adults [12], [13]. Paracetamol toxicity may be attributable to its common availability and the general perception of being extremely safe [12]. Paracetamol poisoning may be as a result of only one acute ingestion or as a result of the repeated use of supratherapeutic amounts [14]. It has been established that the susceptibility to paracetamol toxicity is more common in some individuals than others particularly at lower overdose levels, and lack of medical intervention may affect prognosis [7].

Accidental and intentional paracetamol poisoning has become a serious public health concern [15], [16], and paracetamol poisoning is at the top of medication poisoning list of several countries [17], [18]. According to reports from the American Association for the Study of Liver Diseases, the incidence of paracetamol-induced hepatotoxicity has increased significantly in the past decades [3], and paracetamol is currently the major cause of acute hepatic failure in the United States of America, Britain and in several European nations [3], [19], [20], [21]. Paracetamol poisoning has been reported as the leading cause of liver transplantation in the United States [12]. More than 100,000 calls made to the United States Poison Control Centers, 2600 hospital admissions, 56,000 visits to the emergency departments, and about 500 deaths were attributable to yearly use of paracetamol [3], [22], [23], [24].

### 1.2 Mechanisms of action of acetaminophen

Therapeutically, acetaminophen is used as an analgesic and antipyretic drug. It is not effective as an anti-inflammatory drug. Compared to the NSAIDs known for their inhibition of cyclooxygenase-dependent production of prostaglandins [3], acetaminophen does not have peripheral anti-inflammatory properties and therefore acts primarily within the central nervous system. In the brain, paracetamol can easily cross the blood brain barrier and selectively inhibits the COX pathway without causing similar COX inhibition in peripheral tissues such as the stomach [3], [25]. Centrally, this mechanism of inhibition of cyclooxygenase enzyme by paracetamol is not achieved via direct binding to the enzyme’s active site, rather acetaminophen tends to reduce the active form of COX, making the enzyme inefficient as a catalyst [26]. Recently, it has been suggested that the mechanism of acetaminophen analgesic effect is via its metabolism to N-acylphenolamine (AM404), which then activates the transient receptor potential vanilloid 1 (TRPV1) and cannabinoid 1 (CB1) receptors in the brain. Apart from activating these two receptors in the brain, analgesia also involves their activation in the spinal cord dorsal horn [27], [28]. The mechanism of action of acetaminophen antipyretic activity involve reduction in the oxidative stress that has been implicated in the release of prostaglandin and fever [29], [30], [31], [32].

### 1.3 Mechanisms of acetaminophen hepatotoxicity

When acetaminophen is taken orally, it is absorbed rapidly from the gastrointestinal tract reaching therapeutic levels (in plasma or blood) between 30 min and 2 h. With an overdose, plasma or blood level reaches peak at 4 h, except in situations involving the co-administration of agents which can slow down gastric motility causing a delay in gastric emptying or when the acetaminophen formulation is a sustained-release form [12], [33]. The elimination half-life of acetaminophen is 2 h. This may increase to 17 h in patients with liver dysfunction. Historically, acetaminophen dose for adults is 650–1000 mg taken every 4–6 h, not exceeding 4 g/day, while in children, the dose recommended is 15 mg/kg 6 hourly, up to 60 mg/kg. In the year 2012, the US Food and Drug Administration suggested a maximum adult daily acetaminophen dose of 3 g, not exceeding 650 mg every 6 h, as needed. McNeil Consumer Healthcare, manufacturer of Tylenol brand of paracetamol, has also implemented further reduction of the maximum recommended daily adult dose of its 500 mg tablet to 3 g, and 3.25 g for its 325 mg Tylenol tablet [34]. Ingesting more than 7.5–10 g of acetaminophen over a 24-hour period increases the risk for developing acetaminophen-induced hepatotoxicity. Alternatively, the risk of hepatotoxicity is heightened in patients consuming more than 4 g over 24 h that also have one or more susceptibility risk factors such as malnutrition, chronic use of alcohol, fasting, and use of P450-inducing drugs) [33], [35], [36]. The minimum dose of acetaminophen that can cause toxic effect in children for a single acute ingestion is 150 mg/kg. However, it has been suggested that in children between the ages of 1–6 years who are healthy, this value should be increased to 200 mg/kg due to the less susceptibility to hepatotoxicity from acute acetaminophen toxicity in this age bracket. In this age group, a relatively larger hepatic mass (from the ratio of organ weight to total body weight) and variation in metabolism may contribute to decrease in hepatic bioactivation capacity and more efficient detoxification and elimination of toxic intermediate of acetaminophen [37].

When a therapeutic dose is administered, paracetamol is metabolized primarily (about 90%) through "first pass" metabolism in the liver via glucuronidation and sulfation [3]. Approximately 2% is excreted unchanged in the urine [35]. In addition, a small fraction (5–15%) of paracetamol is metabolized by CYP450 enzymes (mostly the CYP1A2, CYP2E1, CYP3A4, and CYP2A6 isoforms). This reaction results in the production of N-acetyl-p-benzoquinoneimine (NAPQI), a reactive intermediate of very high toxicity [3], [38]. Glutathione reduces NAPQI to nontoxic mercaptate and cysteine compounds, which are then safely excreted by the kidneys or bile [12]. However, when there is an overdose, the primary means of paracetamol metabolism (phase II conjugation) may become saturated, and more of the paracetamol metabolism is diverted into the CYP450 route, resulting in more accumulation of NAPQI [3], [39]. Paracetamol overdose also results in depletion of glutathione stores. A depletion in glutathione level below 30% of normal causes NAPQI binding to hepatic macromolecules resulting in dose-dependent, potentially irreversible hepatic necrosis [3]. The glucuronidation capacity of the liver depends on the stores of carbohydrate such that in malnourished individuals, more paracetamol will be converted to NAPQI. Glutathione stores is also dependent on nutritional status, as malnutrition leads to its depletion [23]. In addition, glutathione level is reduced in alcoholics and HIV patients ([12].

It has been suggested that paracetamol poisoning may cause another episode of tissue injury that does not depend on its metabolism but rather caused by activation of the immune system, particularly the innate immune system [40], [41], [42], [43]. Due to the occurrence of massive hepatocyte death over a period of time, rapid innate immune response is activated. This results in activation of Kupffer cells via toll-like receptors by damage associated molecular patterns (DAMPs) released from necrotic hepatocytes. The Kupffer cells then produce pro-inflammatory cytokines and chemokines that generate a systemic inflammatory reaction which involves migration of myeloid effector cells to the liver [7], [44]. Although controversial, neutrophils have been postulated to cause tissue damage via the formation of reactive oxygen species and the release of proteases [45].

The hepatotoxicity of paracetamol has been established as a major cause of acute hepatic failure [7]. In fatal cases in which paracetamol induce fulminant hepatic failure, the major causes of death are cerebral oedema or sepsis, in the early phase, and multiorgan failure later [46]. Although paracetamol-related fatalities have been mostly attributed to hepatic failure due to massive centrilobular necrosis in association with renal tubular necrosis, some cases of heart toxicity have been reported [47]. Autophagy has been reported to play very important role in paracetamol-induced liver injury. Autophagy is a highly conserved intracellular pathway for degradation aimed at recycling cellular constituents and damaged organelles in response to adverse environmental conditions and stresses as a survival strategy. Pieces of evidence suggest that autophagy is activated in response to paracetamol overdose in specific areas of the liver thereby protecting against paracetamol-induced liver injury [48], [49].

### 1.4 Clinical presentation

Initial symptoms of paracetamol toxicity are often not specific, mild, and do not adequately forecast subsequent liver toxicity [35], [50]. Clinically, paracetamol poisoning is often subdivided into four stages [35], [51].

Stage I: (30 min to 24 h). Individuals may show no symptoms or may have symptoms such as nausea, emesis, malaise, lethargy, diaphoresis, and pallor.

Stage II: (24–72 h). The clinical and laboratory signs of hepatotoxicity and, sometimes, nephrotoxicity become obvious [35]. Symptoms may include vomiting, in addition to pain in the right upper quadrant and hypotension [12], [51]. Sometimes, aminotransferases may rise as early as 8–12 h after paracetamol ingestion in severely poisoned patients [35].

Stage III: During this stage (72–96 h), there may be significant hepatic dysfunction with kidney failure, coagulopathies, jaundice, hyperammonaemia, bleeding diathesis, and hepatic encephalopathy (confusion) [35], [51]. There may be reappearance of Gastrointestinal symptoms. Death is commonly seen at this stage, mainly as a result of multiorgan system failure [52]. Symptoms of severe hepatotoxicity involve plasma ALT and AST levels often greater than 10,000 IU/L, prolongation of the PT or INR, hypoglycaemia, lactic acidosis, and a total bilirubin concentration greater than 4.0 mg/dL [35].

Stage IV (4 days to 3 weeks): This is the recovery phase for patients who survived stage III, usually beginning from day four and completed at about the seventh day after paracetamol overdose. In patients who are seriously ill, recovery can be slower; symptoms and laboratory values may not fall into normal rages for weeks. Histopathological changes in the liver differ from cytolysis to centrilobular necrosis, and restoration of histology may take up to three months after clinical recovery [35].

### 1.5 Diagnosis

Diagnosis of paracetamol poisoning is predicated on serum levels of the drug, even in the absence of symptoms. Serum level above 140 µg/mL at 4 h from ingestion is considered toxic. If the levels fall within toxic range based on the Rumack-Matthew Nomogram, then antidote treatment should commence. The nomogram interprets the blood paracetamol concentration by using plots of concentration against time after overdose [53]. In order to use the nomogram properly, the serum levels must be measured between 4 and 24 h from the time paracetamol was ingested, and only applies to single acute ingestion [54], [55], [56]. However, due to the short half-life of paracetamol (2 h), only few patients with acute liver injury will reveal the parent compound in blood [57], [58]. Blood paracetamol concentration is only a surrogate biomarker of liver toxicity which can result in patient overtreatment; if left untreated, a large population of patients may not have significant hepatic injury [59]. Paracetamol adducts in plasma, are particularly useful for diagnosis of paracetamol ingested at multiple timepoints or greater than 3 days prior to medical care [60], [61]. Urinalysis, EKG, and a metabolic panel may also be needed [12]. Some important differential diagnoses should be taken into consideration when dealing with paracetamol poisoning. They include: hepatorenal disorders, viral hepatitis, pancreatitis, cytomegalovirus infection, gastroenteritis, and peptic ulcer among others [12].

### 1.6 Serum Alanine aminotransferase (ALT) activity, a biomarker of paracetamol liver toxicity

Serum ALT is routinely used in the diagnosis of hepatic injury following paracetamol overdose, and has been validated by years of clinical experience [53], [62]. Evaluation of serum ALT activity is relatively cheap and commonly used in most laboratories in the hospital. At the initial presentation to hospital, patients with paracetamol poisoning who exhibit acute liver failure almost always have an increase in serum ALT activity (probably due to their late report to the hospital), thereafter, they show rapid increases in ALT [63], [64]. This biomarker is useful for identification of patients with fatal liver toxicity that necessitates emergency liver transplantation if they show other symptoms of hepatic failure like coagulopathy, encephalopathy or acute renal injury [53]. In order to confidently exclude hepatotoxicity, at least a 24-hour delay is required between overdose with paracetamol and evaluation of ALT (this may have a negative impact on the patient due to increased length of stay in the hospital, and delay in patient profiling for emergency treatment). An additional drawback with the use of ALT as biomarker of paracetamol poisoning is the likelihood of false-positive results, given that increases in serum ALT activity also occur with several other liver pathologies [63]. Also, increase in serum ALT activity is seen with extreme exercise and rhabdomyolysis as ALT is present in muscle [65].

### 1.7 Upcoming sensitive and specific biomarkers of hepatotoxicity

Prevention of liver toxicity is the critical clinical outcome for treatment of paracetamol poisoning. Hence, there is increasing need for new biomarkers whose primary focus should be on directly reporting liver injury instead of using surrogate markers [53]. Sensitive biomarkers may prove useful in early detection of hepatotoxicity with greater certainty, and equally identify potentially fatal cases of liver injury which cannot be identified by current biomarkers, thereby helping to reduce hospital bed occupancy and adverse effects from unwarranted treatment with antidotes [53]. Researches aimed at bridging the gap between clinical and preclinical studies has led to the development of prospective drug-induced liver injury (DILI) biomarkers [66]. They include biomarkers that are liver specific such as the microRNA-122 [67], [68] or that give information on the mechanism of cell death, for example the high-mobility group box-1 (HMGB1) and keratin-18 (K18) biomarkers [53]. A cohort study in humans has demonstrated evidence for the use of miR-122, HMGB1 and K18 as biomarkers of paracetamol-induced hepatic injury, prognostic evaluation and assessment of outcome for the patient [69], [70].

### 1.8 Treatment of acetaminophen toxicity

Management of acetaminophen toxicity is a function of the time of ingestion of the drug. If the patient is presented within an hour of poisoning with the drug, gastrointestinal decontamination may be carried out. In patients who are conscious, use of activated charcoal may be necessary. However, the use of whole bowel irrigation or orogastric lavage has been reported to be ineffective [12], [71], [72], [73]. Those showing elevated levels of paracetamol should be admitted and treated with the antidote, N-acetyl-cysteine (NAC) [([12]]. The antidote helps in replenishing liver glutathione level, thereby increasing the safe detoxification of N-acetyl-p-benzoquinone imine, NAPQI [74]. NAC also works by preventing NAPQI from binding to macromolecules of the liver, acting as a replacement for glutathione, sulfate precursor, and can reduce NAPQI back to acetaminophen [12]. When given immediately after acetaminophen ingestion (approximately within 8 h), N-acetyl-cysteine is very effective and can prevent significant liver injury [75]. However, the efficacy of N-acetyl-cysteine substantially declines if treatment is delayed [53]. Hence therapeutic effectiveness is highly dependent on when treatment with the antidote is initiated in relation to time of poisoning. N-acetyl-cysteine is therapeutically indicated when serum levels are within range of toxicity using the Rumack-Matthew nomogram; when acetaminophen level is above 10 µg/mL when the time of ingestion is unknown; when a dose of acetaminophen above 140 mg/kg was ingested at time greater than 8 h; and ingestion showing any symptoms of hepatotoxicity [76]. NAC can be given either intravenously or via oral route. The oral formulation has a foul rotten egg odour and taste hence, the intravenous administration is better tolerated by patients, and has been known to reduce the period of hospital stay. Additionally, while the oral formulation requires about 18 doses administered every 4 h, resulting in total treatment time of 72 h, the intravenous route requires only 20 h of therapy, and is the preferred route in pregnant patients, unconscious patients, and in the case of fulminant liver failure [12]. N-acetyl-cysteine administration should still be carried out in patients presenting 24 h after the ingestion of paracetamol as it may enhance survival. Hemodialysis may also be effective, especially when concurrent renal failure exists [12]. Dosage adjustment is not necessary in alcoholics, and redetermination of paracetamol levels is also not required after treatment has started. However, some clinicians administer a loading dose of NAC immediately after hospital arrival if there is an indication (from the patient or family member) that a very large single dose was ingested, pending the determination of blood levels of paracetamol. Then, based on blood levels, the remaining course of NAC treatment is decided upon. Where there is fulminant hepatic failure, N-acetyl-cysteine should be continued beyond the 72-hour period until liver transplant is carried out, patient’s full recovery or death [77], [78]. If diagnoses and therapy are carried out early, mortality from paracetamol poisoning is below 2%. On the other hand, if patient shows up late and with signs of serious hepatic failure, mortality is higher [12]. About 1–3% of patients who developed severe hepatic failure requires liver transplant to save their lives [79], [80], [81]. The overall prognosis depends on parameters such as: Creatinine levels above 3.4 mg/dL, arterial blood pH below 7.3 despite adequate fluid hydration, presentation with grade 3 or 4 encephalopathy, and prothrombin time above 1.8 times the control or an international normalized ratio, INR greater than 6.5 [12]. Generally, children below 6 years have better prognoses than adults, primarily due to their greater ability for detoxification of paracetamol [12]. This work aims at examining the likelihood of paracetamol use at a maximum recommended daily adult dose of 4 g in Nigeria causing more liver toxicity relative to a lower maximum daily adult dose of 3 g emphasized in the United States of America.

## 2 Methods

### 2.1 Searching of data base, search strategy and selection criteria

Online searches were carried out in the databases of PubMed, Google Scholar and MEDLINE for relevant literature using terms such as "paracetamol toxicity," "hepatotoxicity of acetaminophen," "paracetamol and liver diseases in Nigeria," "causes of liver disorders," "liver failure and paracetamol." Further search of related references in PubMed was carried out, in addition to searching the website of the National Agency for Food and Drug Administration and Control (NAFDAC) for updates on paracetamol toxicity. There was no restriction on date of publication. Final search was carried out on October 31, 2021. Further steps involved screening of searched results with their titles and abstracts read for eligibility. Full texts of all eligible publications were obtained with the application of inclusion and exclusion criteria to determine suitable articles used in this review. Findings obtained from the selected studies were summarized in a narrative synthesis.

### 2.2 Inclusion and exclusion criteria

Studies involving human beings, and those involving liver injury or hepatotoxicity in actual human tissue samples, including case reports, mechanistic studies, observational studies and randomized-controlled trials were included. Studies carried out in laboratory animals, and those not published in the English language were excluded.

## 3 Results

### 3.1 Search results

**Fig. 1:**
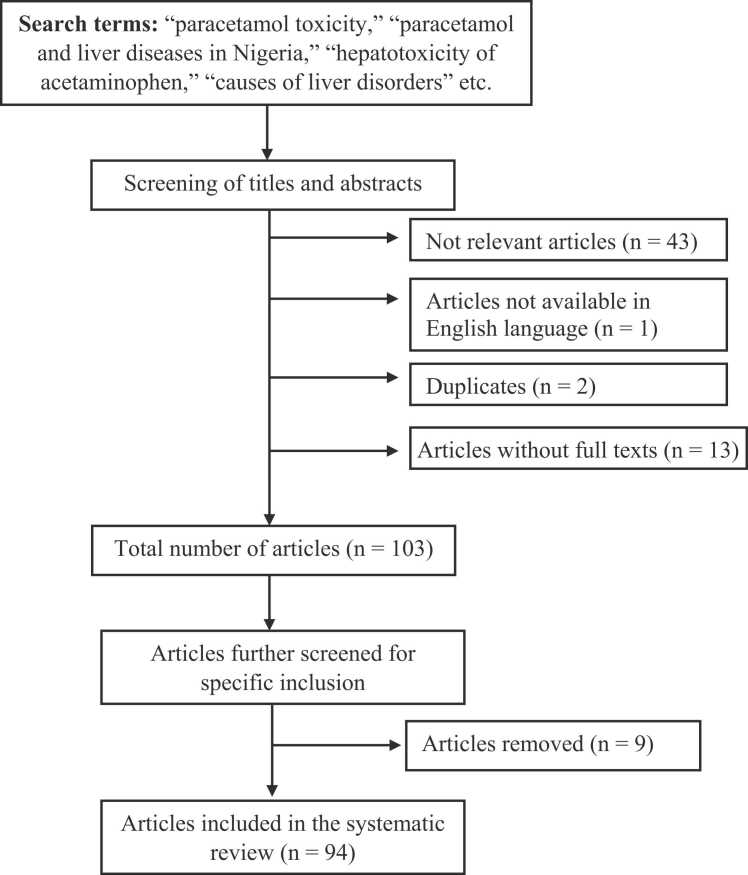
Flow diagram of study selection PRISMA (Preferred Reporting Items for Systematic Reviews and Meta-Analyses).

The initial search returned One hundred and seventy-eight (178) studies. After screening of the abstracts and titles, exclusion of 59 articles was carried out for being irrelevant (n = 43), nonavailability of full texts (n = 13), not available in English (n = 1), duplicates (n = 2). Full texts of the remaining 103 articles were further reviewed, resulting in the exclusion of 9 other publications, thus leaving 94 studies that were used in this review (Fig. 1).

### 3.2 Some incidents and pattern of hepatic diseases in Nigeria

The pattern of liver disease varies from one geographical location to another. It also depends on several factors including genetic, lifestyle, and the environment [82]. Non-Caucasian race has been postulated have higher overall 21-day mortality, which may be a reflection of the differences in accessing healthcare or liver transplantation, or the result of genetic differences in the biotransformation and disposition of paracetamol [19]. In several countries of Africa including Nigeria, there is scarcity of reliable data about the risk factors, hepatic disease admissions, and causes of morbidity and mortality [82]. Deaths due to liver disease in Nigeria reached 60,044 or 3.10% of the total deaths, making Nigeria the second country in the world with age adjusted death rate of 64.44 per 100,000 of population [83].

According to [82], death associated with hepatic disease is very high in Nigeria and is a common cause of hospital admissions. They reported 138 (47.6%) deaths among people with hepatic disease admitted into a major hospital in Ile-Ife, South-West of Nigeria, between 2013 and 2017. They stated that out of a total number of 5155 patients on admission in the hospital, hepatic disorders accounted for about 324 (6.3%) within the 5-year period under review. A breakdown of the report showed that hepatocellular carcinoma (HCC) accounted for 52.8%, followed by 27.2% for liver cirrhosis (LC), metastatic hepatic disease was 4.1%, acute hepatitis (10.38%), autoimmune hepatitis (1.7%), drug-induced liver injury, DILI (0.7%), abdominal tuberculosis (1.4%), liver abscess (1%), and unknown aetiology (1.76%) [82]. Apart from infectious agents, gastrointestinal and liver diseases were also reported to be the major causes of death in a different hospital, South-West of Nigeria [84]. In another report, hepatitis B was implicated as the commonest cause of hepatic disease in Nigeria [85]. In the city of Calabar, south–south zone of Nigeria, another report gave the prevalence rate of 62.3% and 12.3% for hepatitis B and C, respectively, in chronic hepatic disease [86]. Another retrospective study whose aim was to determine the pattern and risk factors of hepatic disease was conducted in a University Teaching Hospital located in Enugu state of Nigeria. The researchers in their report stated that diseases of the liver constituted 7.9% of hospital admissions and hepatocellular carcinoma and liver cirrhosis were responsible for 44.3% and 20.4%, respectively. This study also reported that consumption of alcohol (52.1%), hepatitis B virus (49.4%), roots and herbs consumption (45.5%), smoking (30.1%) are factors that predispose to liver diseases [87].

There are also some reports on the pattern of liver diseases among children in Nigeria. One of them is a retrospective study of children aged less than 16 years, under management for five years (2009–2014) in the department of Paediatric gastroenterology, hepatology and nutrition (PGHAN) clinic and the Emergency paediatric unit of the national hospital located in Abuja, the nation’s capital. The commonest aetiology in this study was reported as viral hepatitis, particularly hepatitis B infection [88]. Infections of hepatitis C were also recorded in this study. These were less common in frequency. The study also reports that some of these paediatric patients had hepatic diseases of unknown cause, with the majority showing complete resolution and were discharged. Another pattern of childhood liver diseases was a 25-year (1986–2010) retrospective study involving all the liver biopsies from children received from the pathology department in the university of Benin teaching hospital, Benin city, south-south Nigeria. In this study, childhood liver diseases constituted 8.9% of all the liver biopsies received at the hospital’s pathology unit. The study reported that the leading causes of childhood chronic liver diseases were inflammatory disorders and tumors (33.3%), cirrhosis/ fibrosis (28.6%) and metabolic diseases (4.8%). Hepatitis (made up of neonatal hepatitis, giant cell hepatitis and viral hepatitis) constituted 33.3% of the cases [89]. In Nigeria and other West African sub-region, the most common aetiology of liver diseases from available reports is hepatitis B virus, with figures between 36.7% and 64% [82], [87], [90]. This increased in prevalence of hepatitis B in Nigeria may be as a result of a low hepatitis B virus vaccination and unwholesome procedures seen with scarification marks and injections administered by unqualified healthcare workers [82]. Sometimes hepatitis could be classified as idiopathic, non-specific or reactive with no identifiable aetiology [88], [91].

### 3.3 The unique case of dangerous and unapproved use of paracetamol in Nigeria

The Nigerian regulatory body known as the National Agency for Food and Drug Administration and Control (NAFDAC) in its Public Alert No. 0023/2019, titled "Alert on Dangerous and Unapproved Use of Paracetamol Tablets in Food Preparation," alerted the population about the dangerous and adverse health effects associated with the use of paracetamol tablets to soften meat used in preparing foods [92]. The agency cautioned members of the public, particularly owners of restaurants to refrain from using paracetamol tablets to soften meat. It emphasized that such practice renders the food unsafe for human consumption, as the paracetamol will be broken down to a toxic material that can cause detrimental health effects such as hepatotoxicity, renal failure, and untimely death with the consumption of such foods.

## 4 Discussion

Paracetamol may be more harmful than Nigerian users think, since even at a lower maximal daily adult dose level of 3 g recommended in the United States of America, paracetamol remains the main culprit in liver failure and liver transplant. Also in the United States, paracetamol was reported to increase the risk for liver transplantation as a result of acute liver injury in non-overdose levels by three folds, and seven-folds in overdose levels of exposure [93]. Paracetamol overdose was also responsible for 97% of all incidents of drug overdoses associated with acute hepatic failure [94].

In Nigeria, most formulations of paracetamol, especially the solid dosage forms, which are frequently prescribed or obtained over-the-counter come in 500 mg strength and taken at 1000 mg every 6–8 h, or on as 'when needed' basis by adults. The famous physician, Paracelsus (1493–1541) credited, "The Father of Modern Toxicology", said: "What is there that is not poison? All things are poison and nothing is without poison. Solely the dose determines that a thing is not a poison." By suggesting that a 'lower dose' prevents adverse effects, Paracelsus had laid the foundation for the today's differentiation of the terms risk and hazard [95]. This is important given the report that paracetamol is hepatotoxic in a dose-dependent fashion and is implicated in almost 50% of all cases of acute hepatic failure in the United States, the United Kingdom and in other western nations [96], [97]. As an additional precaution to further stem the tide of paracetamol hepatotoxicity, some National Health Service organizations in the United Kingdom have suggested that a lower dose of paracetamol taken orally should be used in those with body weights less than 50 kg [23]. Historically, the maximum dose of paracetamol to be taken daily by adults is 4 g, while in children below 12 years and/or below 50 kg, the maximum dose given daily is 75 mg/kg. However, producers of the brand of acetaminophen known as Tylenol (McNeil Consumer Healthcare) have reduced the maximum dose recommended for adults of its 500 mg tablet formulation to 3 g, and that of its 325 mg tablet to 3.25 g daily [37].

High rates of accidental and intentional paracetamol poisoning, especially in children calls for increased public awareness of the risks associated with paracetamol overdose [15], [98], [99], especially at the current therapeutic dosage regimen used in Nigeria. There is paucity of published literature on the subject matter of paracetamol poisoning which is an indication of dearth of case reports by emergency departments in Nigeria. This erroneously leads to under reporting of liver disorders that may be caused by paracetamol poisoning, or categorizing such disorders as being due to unknown aetiologies. In Nigeria, diagnosis of liver diseases involved using clinical symptoms, CT scans, ultrasonography of the abdomen, liver function tests, viral hepatitis serology, and hepatic biopsies [82]. Determination of blood paracetamol concentration was rarely reported, thus excluding acetaminophen poisoning as one of the likely causes of hepatic disorders in Nigeria. This may be attributable to the lack of adequate investigative capacity for assay of blood paracetamol concentration due to lack of advanced laboratories and financial constraints. The aetiology of liver injury is a major determinant of transplant-free survival in patients with acute hepatic failure [20]. About 75% of individuals with paracetamol-induced acute hepatic failure attain full recovery, in comparison with about 40% of patients with acute hepatic failure from other causes. Apart from paracetamol, other causes with better transplant-free survival include: those with hepatitis A (56% transplant-free survival), ischaemic liver injury (74%), and pregnant women with gestational cholestasis (83%). On the flip side, etiologies with low transplant-free survival include hepatitis B virus (26%), drug-related hepatic injury (41%), autoimmunity (25%), and unknown causes (33%) [57].

Several factors may contribute to enhanced paracetamol hepatotoxic potentials. In Nigeria, several over-the -counter and prescription medications contain paracetamol in combination with other active ingredients. The paracetamol contents in these products are variable, and maybe up to 500 mg per unit dose. For example, the cold medication market under the brand name, Procold® contains 500 mg paracetamol in combination with pseudoephedrine HCl 30 mg, and chlorpheniramine maleate 2 mg. A report from two investigators, Ejeikwu and Folashade [2] while evaluating the risk perception of paracetamol usage by the University of Jos undergraduate students in north-central Nigeria, stated that most of the students did not know that simultaneous use of paracetamol and Procold® constitutes any health hazard. Infants and children are particularly vulnerable to paracetamol overdoses due differences in dosing schedules and the multiplicity of preparations with varying strengths of paracetamol [11], [100]. The simplest way to unknowingly overdose children with paracetamol is to give it simultaneously with many colds, cough, and teething drugs which may all individually contain different strengths of paracetamol [100].

There is a rise in report of fatal overdoses involving acetaminophen combination products to the United States poison centers [46] where acetaminophen is commonly combined with opioids, antihistamines, dextromethorphan, decongestants, and caffeine [46], [101]. The American Association of Poison Control Centers reported that acetaminophen combination products were associated with 48% of paracetamol-related fatalities [102]. The combination products responsible for most fatalities are those containing acetaminophen and opioids. However, after thorough investigation of several deaths arising from ingestion of acetaminophen combination products reported to the United States Poison Centers, it was affirmed that acetaminophen was actually responsible for patients’ death, and not the combination products [46]. After the 2009 Advisory Committee meeting, the United States Food and Drug Administration (FDA) released a statement in January 2011 that any prescription formulation of acetaminophen combined with other drugs (particularly oxycodone- or hydrocodone-acetaminophen) marketed after January 2014 should contain only 325 mg per tablet as against the previous formulations that contained 500, 650 or 750 mg of acetaminophen per tablet [103]. In addition to this specification currently in effect in the United States, current package labeling must include ‘severe liver injury’ warning as a likely outcome, if more than 4.0 g is taken within 24 h, or with other acetaminophen-containing substances or alcohol. In Nigeria, formulations containing combinations of paracetamol and narcotic analgesics in excess of 325 mg of paracetamol per tablet are common, for example, Co-codamol 8/500 mg tablet which contains 8 mg codeine phosphate and 500 mg acetaminophen. The use of such drugs may constitute a major source of paracetamol toxicity, especially in individuals who may develop tolerance and dependence as a result of the narcotic ingredients in these formulations, and may choose to arbitrarily increase the dose. The problem with these combination formulations tends from the fact that majority of those who take them may not have the requisite knowledge, or are reluctant to read the inserts or packaging labels to know whether these preparations contain paracetamol. Additionally, many users have no information or may not understand the ‘maximum recommended daily dose’ concept, and are unaware of the likelihood for developing liver toxicity with increased dose, despite being cautioned and appropriate dose recommendations on the drug label or package inserts [3].

Another factor that may contribute to paracetamol toxicity in Nigeria is the over-the-counter (OTC) status of paracetamol. Just like in many other countries, acetaminophen is not a controlled medication in Nigeria and users do not require a prescription from physicians before getting it. This has resulted in increased self-medication and indiscriminate use of this drug, leading to possible health hazards among users that are likely to be uninformed of their possible adverse effects. Self-medication is commonly practiced and may be the cause of several cases of paracetamol poisoning [3]. Apart from the usual acquisition of several drugs over-the-counter in the pharmacy or from patent medicine vendors, self-medication practice and indiscriminate use of drugs are seen with the occasional use of an ‘endorsed’ medication or the patient going for an unused or remaining drug purchased for treatment during past illnesses [104]. The study conducted by Ejeikwu and Folashade [2] among undergraduate students in a Nigerian university reported that a significant population of participating students had used paracetamol on 40 or more occasions in their lifetime, and majority of them had no understanding that self-medication with paracetamol can be harmful. In addition, most of the students had no knowledge of paracetamol contraindications in hepatitis and liver cirrhosis. On the whole, many of these individuals exhibited poor risk perception of paracetamol use. Salami and Adesanwo [105] who examined self-medication practice among mothers of children below 5 years in Ibadan, south-west Nigeria had reported that 53.4% of mothers employed self-medication as their first action when the children became ill.

Another study was aimed at identifying the several patterns of self-medication seen with caregivers to children below 5 years in Osun and Lagos states of Nigeria. In the urban environment, it was reported that pattern of self-medication tended towards the use of majorly paracetamol and herbal preparations. Paracetamol was predominantly used among the caregivers to the under-five children to treat fever. This was attributed to the existence of hospitals, pharmacies, and patent medicine shops in the urban area [106]. Another side of the study showed that participants in the rural environment had inadequate or no access to medical care as they were non-existent. Hence, the engaged in self-medication using traditionally produced herbal concoctions, some western medicines that contain paracetamol in combination with other ingredients, and faith-based practices which involved prayer and fasting, use of spiritual water, and anointing oil.

In another scenario, an observational prospective study involving 231 children aged between 6 weeks and 16 years was conducted at the paediatric outpatient clinic of the University of Nigeria Teaching Hospital, Enugu, south-east Nigeria, from June to November 2011, to examine paediatric paracetamol use and/or misuse patterns. These researchers reported that most of the paediatric paracetamol usage were self-prescribed, with possibility of misuse and overdosing. These observations were attributed to readily accessibility and availability of paracetamol, illiteracy and poverty [100]. Majority of the paediatric users 175 (75.6%) were given paracetamol at home before being taken to the clinic and most of the caregivers (71.2%) used previous experiences to determine the dose of paracetamol. Although paracetamol use is generally safe in therapeutic doses, cases of liver toxicity have been seen with recommended dosages among the paediatric population in developing countries [107]. Other factors that predispose to chronic paracetamol poisoning include repeated ingestion of elevated doses, as well as repeated ingestion of recommended doses within short time intervals [108].

Another important factor that may predispose some individuals to paracetamol toxicity is poor nutritional status [7]. Malnutrition is a major public health issue, in developing nations, including Nigeria, where 90% of the world's malnourished children live [109], [110]. Malnutrition is seen as either micronutrient malnutrition or protein-energy malnutrition [111]. Malnutrition (acute and chronic) may result in an increased tendency to paracetamol poisoning, particularly if multiple doses are ingested concurrently [23], [112]. Incidents of compromised nutritional status can also be seen from fasting for a long time, eating disorders, HIV, cystic fibrosis, gastroenteritis and chronic alcohol intake [37]. It has been postulated that a malnourished state depletes hepatic glutathione, thereby reducing the capacity for detoxification of NAPQI, leading to increased risk of toxicity [23], [113]. Hepatic glucuronidation is usually dependent upon hepatic carbohydrate reserves. In the fasting or undernourished state, glucuronidation of paracetamol is reduced, leading to enhanced microsomal metabolism and increased production of the toxic NAPQI metabolite [35], [113]. Malnutrition may be a direct result of hepatic diseases, with overweight and obesity as predisposing conditions for the liver disorders [88]. From demographics, males and children with lower socioeconomic statuses are more vulnerable to hepatic diseases [114], [115], [116]. The fact that causality has not been proven has brought controversies surrounding the role of malnourished state as a predisposing factor in paracetamol poisoning [23], [117], [118].

Miscalculation of dosage and inadequate knowledge of timing of dosage have also been implicated as one of the reasons behind some cases of severe liver injury or even deaths from acetaminophen usage [15]. Acetaminophen dosage forms include the oral dosage (tablets, capsules, syrup, or drops), injectable, and suppositories (for rectal use). Oral route is preferable in children by caregivers. The liquid form is commonly used for being more precise in right dose estimation [15]. Most parents have been reported to use syringes to measure the dose of acetaminophen syrup, while some reportedly used inaccurate measures such as teaspoons, tablespoons, and regular kitchen spoons [15], [119]. This may be another reason for the increased incidents of incorrect dosing of acetaminophen in the paediatric population by caregivers [15]. A study which evaluated patterns of acetaminophen use and misuse among children in Enugu, South-East Nigeria had reported that apart from only two children who were given injectable acetaminophen, oral route was preferred 228 (98.9%). The oral dosage forms used were 49.2% for tablet, 38.7% for syrup, while 28 12% were given both syrup and tablet [100].

It has been suggested from case reports and retrospective studies that chronic intake of alcohol tends to increase paracetamol poisoning risk, even with therapeutic drug doses [23], [120], [121]. Concerns around increased susceptibility to paracetamol poisoning from chronic intake of alcohol was the rationale behind the consideration for use of NAC in therapy of paracetamol poisoning where risk stratification was recommended [122]. Chronic consumption of alcohol tends to increase susceptibility to paracetamol poisoning due to CYP2E1 induction, resulting in decrease in hepatic glutathione stores that culminates in reduced detoxification by NAPQI or reduction in the glucuronidation pathway [23], [120], [121], [123], [124]. In addition to higher NAPQI generation by CYP2E1 induction, ethanol is also metabolized to a free radical intermediate by this iso-enzymatic form of cytochrome P450 (CYP2E1). The free radicals generated bind to microsomal protein, especially the CYP2E1 itself, resulting in lipid peroxidation which may cause ethanol-induced hepatotoxicity [125]. However, prospective studies in controlled clinical trials have found no relationship with chronic intake of alcohol and increased susceptibility to paracetamol poisoning at therapeutic doses [59], [117], [123], [126], [127], [128], [129].

In addition to the aforementioned, some prescription drugs and over-the-counter medications are known to predispose a patient to acetaminophen hepatotoxicity. They include opioids, anti-seizure drugs, anti-tuberculosis drugs, dietary supplements, such as garlic, St. John’s wort, and germander, via their effects on CYP450 metabolism ([130]; Wang et al., 2016). The risk for hepatotoxicity is also affected by age, with advanced age (above 40 years) associated with a higher risk of acute hepatic failure, need for liver transplant, and death from acetaminophen overdose [131]. There is paucity of evidence suggesting pregnancy as a risk factor for acetaminophen toxicity [132]. However, usage of paracetamol during pregnancy should be monitored carefully, since acetaminophen overdose is common during pregnancy, and the resultant toxicity may lead to significant morbidity and mortality for the pregnant mother and fetus [130], [133].

## 5 Conclusion

It is rather surprising that in Nigeria, there is paucity of studies involving paracetamol as an etiologic agent in the spectrum of liver disorders given its status as the most used drug and pain medication. Evaluation of liver diseases in Nigeria was predicated mostly on constellation of clinical features, and limited laboratory investigations that most often did not include blood paracetamol level determination. In addition, literature reports on intentional and unintentional paracetamol poisoning episodes in this world's most populous black nation are scanty. In Nigeria, rampant use of the commonly available 500 mg strength of acetaminophen coupled with the tendency to surpass the recommended maximum dose of 3 g per day, may increase its risk for hepatotoxicity than observed in the United States of America known for emphasizing lower dose regime of the drug. There may be need to investigate the effectiveness of paracetamol at lower dose level, as well as its toxicity at the current dose regime used in Nigeria. Community and hospital Pharmacists should counsel individuals taking paracetamol not to surpass the new maximum recommended adult daily dose, to take paracetamol when absolutely needed, and emphasize its hepatotoxic potentials. In Nigeria, the regulatory agency, NAFDAC should consider a reduction in the content of paracetamol in fixed-dose combinations to 325 mg, in addition to reducing the total number of tablets or capsules of each unit dispensed pack in these combination products.

## CRediT authorship contribution statement

**SJO:** Writing – original draft. **CNA, IC-N** and **JEM:** Writing – review & editing. **OEO:** Conceptualization, Writing – review & editing.

## Declaration of Competing Interest

The authors declare that they have no known competing financial interests or personal relationships that could have appeared to influence the work reported in this paper.

## References

[1] Bentley E., Mackie I.C. (1995). Trends in prescriptions of paracetamol for children. BMJ.

[2] Ejeikwu T.M., Folashade W. (2019). Risk perception of paracetamol use among undergraduate students of University of Jos. Open Access Libr. J..

[3] Ghanem C.I., María J.P., Manautou J.E., Mottino A.D. (2016). Acetaminophen: from liver to brain: new insights into drug pharmacological action and toxicity. Pharmacol. Res..

[4] Pandolfini C., Bonati M. (2005). A literature review on off-label drug use in children. Eur. J. Pediatr..

[5] Katzung B.G., Susan B.M., Trevor A.J. (2009). Basic and Clinical Pharmacology.

[6] Rajaram P., Subramanian R. (2018). Management of acute liver failure in the intensive care unit setting. Clin. Liver Dis..

[7] Athersuch T.J., Antoine D.J., Boobis A.R., Coen M., Daly A.K., Possamai L., Nicholson J.K., Wilson I.D. (2018). Paracetamol metabolism, hepatotoxicity, biomarkers and therapeutic interventions: a perspective. Toxicol. Res..

[8] Jasani B., Weisz D.E., McNamara P.J. (2018). Evidence-based use of acetaminophen for hemodynamically significant ductus arteriosus in preterm infants. Semin. Perinatol..

[9] Cranswick N., Coghlan D. (2000). Paracetamol efficacy and safety in children: the first 40 years. Am. J. Ther..

[10] Suzan S.M., Thomas G., Wayne F., Robert R.T. (2005). Pediatrics: Just the Facts.

[11] Utpal K.S., Ramesh K.P., Sachdev H.P.S., Panna C., Arvind B., Krishan C., Siddarth R., Ramesh K.P. (2004). Principles of Pediatric and Neonatal Emergencies.

[12] S. Agrawal, B. Khazaeni, Acetaminophen Toxicity, in: StatPearls (Internet), Treasure Island (FL), StatPearls Publishing (Updated 2021 Jul 18), 2021. 〈https://www.ncbi.nlm.nih.gov/books/NBK441917/#_NBK441917_pubdet_〉. (Accessed 11 October 2021).

[13] Penna A., Buchanan N. (1991). Paracetamol poisoning in children and hepatotoxicity. Br. J. Clin. Pharmacol..

[14] Ammar A.F., Alkhuzaee M., Alsulami N., Bushah A., Almalki A., Torkistani A. (2019). Mini review study of acetaminophen overdose poisoning and associated factors in cases from poison. Austin. Biochem..

[15] Daifallah A., Jabr R., Al-Tawil F., Elkourdi M., Salman Z., Koni A., Samara A., Al-Jabi S.W., Zyoud S.H. (2021). An assessment of parents’ knowledge and awareness regarding paracetamol use in children: a cross-sectional study from Palestine. BMC Public Health.

[16] Manthripragada A.D., Zhou E.H., Budnitz D.S., Lovegrove M.C., Willy M.E. (2011). Characterization of acetaminophen overdose-related emergency department visits and hospitalizations in the United States. Pharmacoepidemiol. Drug Saf..

[17] Fontana R.J. (2008). Acute liver failure including acetaminophen overdose. Med. Clin. N. Am..

[18] Hawkins L.C., Edwards J.N., Dargan P.I. (2007). Impact of restricting paracetamol pack sizes on paracetamol poisoning in the United Kingdom: a review of the literature. Drug Saf..

[19] Puri P., Lee W.M., Fontana R.J., Kim N., Durkalski V., McGuire B.M., Liou I., Pezzia C., Todd Stravitz R. (2020). Alcohol consumption is associated with the severity and outcome of acute liver injury/failure. Liver Int..

[20] Reuben A., Tillman H., Fontana R.J. (2016). Outcomes in adults with acute liver failure between 1998 and 2013: an observational cohort study. Ann. Intern. Med..

[21] Bernal W., Wendon J. (2013). Acute liver failure. N. Engl. J. Med..

[22] Bronstein A.C., Spyker D.A., Cantilena L.R., Green J., Rumack B.H., Heard S.E. (2007). The 2006 Annual Report of the American Association of Poison Control Centers’ National Poison Data System (NPDS). Clin. Toxicol..

[23] Caparrotta T.M., Antoine D.J., Dear J.W. (2018). Are some people at increased risk of paracetamol-induced liver injury? A critical review of the literature. Eur. J. Clin. Pharmacol..

[24] Chiew A.L., Domingo G., Buckley N.A., Stathakis P., Ress K., Roberts D.M. (2020). Hepatotoxicity in a child following an accidental overdose of liquid paracetamol. Clin. Toxicol..

[25] Engstrom R.L., Wilhelms D.B., Eskilsson A., Vasilache A.M., Elander L., Engblom D., Blomqvist A. (2013). Acetaminophen reduces lipopolysaccharide-induced fever by inhibiting cyclooxygenase-2. Neuropharmacology.

[26] Boutaud O., Aronoff D.M., Richardson J.H., Marnett L.J., Oates J.A. (2002). Determinants of the cellular specificity of acetaminophen as an inhibitor of prostaglandin H(2) synthases. Proc. Natl. Acad. Sci. USA.

[27] De Gregorio D., McLaughlin R.J., Posa L., Ochoa-Sanchez R., Enns J., Lopez-Canul M. (2019). Cannabidiol modulates serotonergic transmission and reverses both allodynia and anxiety-like behavior in a model of neuropathic pain. Pain.

[28] Ohashi N., Kohno T. (2020). Analgesic effect of acetaminophen: a review of known and novel mechanisms of action. Front. Pharmacol..

[29] Riedel W., Lang U., Oetjen U., Schlapp U., Shibata M. (2003). Inhibition of oxygen radical formation by methylene blue, aspirin, or alpha-lipoic acid, prevents bacterial-lipopolysaccharide-induced fever. Mol. Cell Biochem..

[30] Tripathy D., Grammas P. (2009). Acetaminophen protects brain endothelial cells against oxidative stress. Microvasc. Res..

[31] Hou C.C., Lin H., Chang C.P., Huang W.T., Lin M.T. (2011). Oxidative stress and pyrogenic fever pathogenesis. Eur. J. Pharmacol..

[32] Maharaj H., Maharaj D.S., Daya S. (2006). Acetylsalicylic acid and acetaminophen protect against oxidative neurotoxicity. Metab. Brain Dis..

[33] Ye H., Nelson L.J., Gómez Del Moral M., Martínez-Naves E., Cubero F.J. (2018). Dissecting the molecular pathophysiology of drug-induced liver injury. World J. Gastroenterol..

[34] S.E. Farrell, L. Germaine, G.L. Defendi, What are the recommended maximum daily dosages of acetaminophen in adults and children? Medscape, 2020. 〈https://www.medscape.com/answers/820200–27207/what-are-the-recommended-maximum-daily-dosages-of-acetaminophen-in-adults-and-children〉. (Accessed 12 December 2021).

[35] M.J. Burns, S.L. Friedman, A.M. Larson, Acetaminophen (Paracetamol) Poisoning in Adults: Pathophysiology, Presentation, and Diagnosis, 2011. 〈https://somepomed.org/articulos/contents/mobipreview.htm?28/29/29137〉. (Accessed 22 October 2021).

[36] Lewis R.K., Paloucek F.P. (1991). Assessment and treatment of acetaminophen overdose. Clin. Pharm..

[37] S.E. Farrell, G.L. Defendi, Acetaminophen toxicity, Medscape, 2021. 〈https://emedicine.medscape.com/article/820200-overview#a4〉. (Accessed 31 October 2021).

[38] Corcoran G.B., Mitchell J.R., Vaishnav Y.N., Horning E.C. (1980). Evidence that acetaminophen and N-hydroxyacetaminophen form a common arylating intermediate, N-acetyl-p-benzoquinoneimine. Mol. Pharmacol..

[39] Zaher H., Buters J.T., Ward J.M., Bruno M.K., Lucas A.M., Stern S.T., Cohen S.D., Gonzalez F.J. (1998). Protection against acetaminophen toxicity in CYP1A2 and CYP2E1 double-null mice. Toxicol. Appl. Pharmacol..

[40] Imaeda A.B., Watanabe A., Sohail M.A., Mahmood S., Mohamadnejad M., Sutterwala F.S., Flavell R.A., Mehal W.Z. (2009). Acetaminophen-induced hepatotoxicity in mice is dependent on Tlr9 and the Nalp3 inflammasome. J. Clin. Investig..

[41] Kubes P., Mehal W.Z. (2012). Sterile inflammation in the liver. Gastroenterology..

[42] Lawson J.A., Farhood A., Hopper R.D., Bajt M.L., Jaeschke H. (2000). The hepatic inflammatory response after acetaminophen overdose: role of neutrophils. Toxicol. Sci..

[43] Liu Z.X., Han D., Gunawan B., Kaplowitz N. (2006). Neutrophil depletion protects against murine acetaminophen hepatotoxicity. Hepatology.

[44] Krenkel O., Mossanen J.C., Tacke F.C. (2014). Immune mechanisms in acetaminophen-induced acute liver failure. Hepatobiliary Surg. Nutr..

[45] Marques P.E., Amaral S.S., Pires D.A., Nogueira L.L., Soriani F.M., Lima B.H., Lopes G.A., Russo R.C., Avila T.V., Menezes G.B. (2012). Chemokines and mitochondrial products activate neutrophils to amplify organ injury during mouse acute liver failure. Hepatology.

[46] Tittarelli R., Pellegrini M., Scarpellini M.G., Marinelli E., Bruti V., di Luca N.M., Busardò F.P., Zaami S. (2017). Hepatotoxicity of paracetamol and related fatalities. Eur. Rev. Med. Pharmacol. Sci..

[47] Singer P.P., Jones G.R., Bannach B.G., DenmarK L. (2007). Acute fatal acetaminophen overdose without liver necrosis. J. Forensic Sci..

[48] Chao X., Wang H., Jaeschke H., Ding W. (2018). Role and mechanisms of autophagy in acetaminophen-induced liver injury. Liver Int..

[49] Parzych K.R., Klionsky D.J. (2014). An overview of autophagy: morphology, mechanism, and regulation. Antioxid. Redox Signal..

[50] McBride P.V., Rumack B.H. (1992). Acetaminophen intoxication. Semin. Dial..

[51] Saccomano S.J. (2019). Acute acetaminophen toxicity in adults. Nurse Pract..

[52] Bessems J.G., Vermeulen N.P. (2001). Paracetamol (acetaminophen)-induced toxicity: molecular and biochemical mechanisms, analogues and protective approaches. Crit. Rev. Toxicol..

[53] Dear J.W., Antoine D.J. (2014). Stratification of paracetamol overdose patients using new toxicity biomarkers: current candidates and future challenges. Expert Rev. Clin. Pharmacol..

[54] Radke J.B., Algren D.A., Chenoweth J.A., Owen K.P., Ford J.B., Albertson T.E., Sutter M.E. (2018). Transaminase and creatine kinase ratios for differentiating delayed acetaminophen overdose from rhabdomyolysis. West J. Emerg. Med..

[55] Levine M., Stellpflug S.J., Pizon A.F., Peak D.A., Villano J., Wiegand T., Dib C., Thomas S.H. (2018). Hypoglycemia and lactic acidosis outperform King’s College criteria for predicting death or transplant in acetaminophen toxic patients. Clin. Toxicol..

[56] McGill M.R., Jaeschke H. (2018). Biomarkers of drug-induced liver injury: progress and utility in research, medicine, and regulation. Expert Rev. Mol. Diagn..

[57] Stravitz R.T., Lee W.M. (2019). Acute liver failure. Lancet.

[58] Leventhal T.M., Gottfried M., Olson J.C., Subramanian R.M., Hameed B., Lee W.M. (2019). Acetaminophen is undetectable in plasma from more than half of patients believed to have acute liver failure due to overdose. Clin. Gastroenterol. Hepatol..

[59] Prescott L.F. (2001). Paracetamol (Acetaminophen): a Critical Bibliographic Review (Revised).

[60] Roberts D.W., Lee W.M., Hinson J.A. (2017). An immunoassay to rapidly measure acetaminophen protein adducts accurately identifies patients with acute liver injury or failure. Clin. Gastroenterol. Hepatol..

[61] Davern T.J., James L.P., Hinson J.A. (2006). Measurement of serum acetaminophen-protein adducts in patients with acute liver failure. Gastroenterology.

[62] Senior J.R. (2012). Alanine aminotransferase: a clinical and regulatory tool for detecting liver injury-past, present, and future. Clin. Pharmacol. Ther..

[63] Al-Hourani K., Mansi R., Pettie J. (2013). The predictive value of hospital admission serum alanine transaminase activity in patients treated for paracetamol overdose. QJM.

[64] Green T.J., Sivilotti M.L., Langmann C. (2010). When do the aminotransferases rise after acute acetaminophen overdose?. Clin. Toxicol..

[65] Zhang Y., Jia Y., Zheng R. (2010). Plasma microRNA-122 as a biomarker for viral-, alcohol-, and chemical-related hepatic diseases. Clin. Chem..

[66] Antoine D.J., Lewis P.S., Goldring C.E., Park B.K. (2013). Are we closer to finding biomarkers for identifying acute drug-induced liver injury?. Biomark. Med..

[67] Matheis K., Laurie D., Andriamandroso C. (2011). A generic operational strategy to qualify translational safety biomarkers. Drug Discov. Today.

[68] Moggs J., Moulin P., Pognan F. (2012). Investigative safety science as a competitive advantage for pharma. Expert Opin. Drug Metab. Toxicol..

[69] Starkey L.P.J., Dear J., Platt V. (2011). Circulating microRNAs as potential markers of human drug-induced liver injury. Hepatology.

[70] Antoine D.J., Jenkins R.E., Dear J.W. (2012). Molecular forms of HMGB1 and keratin-18 as mechanistic biomarkers for mode of cell death and prognosis during clinical acetaminophen hepatotoxicity. J. Hepatol..

[71] Imani F., Motavaf M., Safari S., Alavian S.M. (2014). The therapeutic use of analgesics in patients with liver cirrhosis: a literature review and evidence-based recommendations. Hepat. Mon..

[72] Janssen J., Singh-Saluja S. (2015). How much did you take? Reviewing acetaminophen toxicity. Can. Fam. Physician.

[73] Alempijevic T., Zec S., Milosavljevic T. (2017). Drug-induced liver injury: Do we know everything?. World J. Hepatol..

[74] Ferner R.E., Dear J.W., Bateman D.N. (2011). Management of paracetamol poisoning. BMJ.

[75] Waring W.S. (2012). Criteria for acetylcysteine treatment and clinical outcomes after paracetamol poisoning. Expert Rev. Clin. Pharmacol..

[76] Fukumoto M. (2010). Are nomograms available for the treatment of acetaminophen poisoning? Limitation of Rumack & Matthew nomogram for evaluation of hepatotoxicity. Chudoku Kenkyu.

[77] Yesil Y., Ozdemir A.A. (2018). Evaluation of the children with acute acetaminophen overdose and intravenous N-acetylcysteine treatment. Pak. J. Med. Sci..

[78] Muñoz Romo R., Borobia Pérez A.M., Muñoz M., Carballo Cardona C., Cobo Mora J., Carcas Sansuán A.J. (2018). Efficient diagnosis and treatment of acute paracetamol poisoning: cost-effectiveness analysis of approaches based on a hospital toxicovigilance program. Emergencias.

[79] Yoon E., Babar A., Choudhary M., Kutner M., Pyrsopoulos N. (2016). Acetaminophen-induced hepatotoxicity: a comprehensive update. J. Clin. Transl. Hepatol..

[80] Chiew A.L., Gluud C., Brok J., Buckley N.A. (2018). Interventions for paracetamol (acetaminophen) overdose. Cochrane Database Syst. Rev..

[81] Frischknecht J. (2013). Order in the house. Setting standards for treatment of acetaminophen toxicity. Adv. NPs Pas..

[82] Adekanle O., Ijarotimi O., Obasi E., Anthony-Nwojo N.G., Ndububa D.A. (2020). A Southwest Nigerian tertiary hospital 5-year study of the pattern of liver disease admission. Niger. J. Gastroenterol. Hepatol..

[83] Agada S.A., Odama R.I., Kenechukwu C.O., Okang S.O., Ezeh C.O. (2020). Epidemiology of chronic liver disease in Nigeria: a review. Asian J. Adv. Med. Sci..

[84] Adekanle O., Olatunde I.O., Abdul-lateef A.R. (2008). Causes and pattern of death in a tertiary health institution in South Western Nigeria. Niger. Postgrad. Med. J..

[85] Berinyuy B.E., Alawode R.A., Mohammed A.B., Babalola B.S. (2019). Prevalence of hepatitis B virus in Nigeria: review update. Ann. Public Health Epidemiol..

[86] Kooffreh-Ada M., Okpara H., Oku A., Ikekwaba P.A. (2015). Risk factors of chronic liver disease amongst patients receiving care in a gastroenterogy practice in Calabar. IOSR J. Dent. Sci..

[87] Nwokediuko S.C., Osuala P.C., Uduma U.V., Alaneme A.K., Onwuka C.C., Mesigo C. (2013). Pattern of liver disease admissions in a Nigerian tertiary hospital. Niger. J. Clin. Pract..

[88] Ahmed P.A., Ulonnam C.C., Mohammed-Nafiu R., Ballong J., Nwankwo G. (2016). Pattern of liver diseases among children attending the National Hospital Abuja, Nigeria. Niger. J. Paed..

[89] Nnadi I.G., Olu-Eddo A.N. (2020). Childhood liver diseases in Nigeria, a 25 (1986-2010) years histopathological study. J. Biomed. Sci. Res..

[90] Iloh G.U., Ikwudinma A.O. (2013). Sero-epidemiology of hepatitis B surface antigenaemia among adult Nigerians with clinical features of liver diseases attending a primary care clinic in a resource constrained setting of Eastern Nigeria. N. Am. J. Med. Sci..

[91] Ahmad M., Afzal S., Roshan E., Mubarik A., Bano S., Khan S.A. (2005). Usefulness of needle biopsy in the diagnosis of paediatric liver disorders. JPMA.

[92] National Agency for Food and Drug Administration and Control, NAFDAC., 2019. Public Alert No. 0023/2019 – Alert on Dangerous and Unapproved Use of Paracetamol Tablets in Food Preparation. 〈https://somepomed.org/articulos/contents/mobipreview.htm?28/29/29137〉. (Accessed 31 October 2021).

[93] Gulmez S.E., Larrey D., Pageaux G.P., Lignot S., Lassalle R., Jové J. (2013). Transplantation for acute liver failure in patients exposed to NSAIDs or paracetamol (acetaminophen): the multinational case-population SALT study. Drug Saf..

[94] Gulmez S.E., Larrey D., Pageaux G.P., Bernuau J., Bissoli F., Horsmans Y. (2015). Liver transplant associated with paracetamol overdose: results from the seven-country SALT study. Br. J. Clin. Pharmacol..

[95] Grandjean P. (2016). Pracelsus revisited: The dose concept in a complex world. Basic Clin. Pharmacol. Toxicol..

[96] Jaeschke H. (2015). Acetaminophen - dose-dependent drug hepatotoxicity and acute liver failure in patients. Dig. Dis..

[97] Lee W.M. (2012). Acute liver failure. Semin. Respir. Crit. Care Med..

[98] Kominek K., Pawłowska-Kamieniak A., Mroczkowska-Juchkiewicz A., Krawiec P., Pac-Kożuchowska E. (2015). Intentional and accidental paracetamol poisoning in childhood - a retrospective analysis. Postepy. Hig. Med. Dosw..

[99] Mund M.E., Quarcoo D., Gyo C., Bruggmann D., Groneberg D.A. (2015). Paracetamol as a toxic substance for children: aspects of legislation in selected countries. J. Occup. Med. Toxicol..

[100] Obu H.A., Chinawa J.M., Ubesie A.C., Eke C.B., Ndu I.K. (2012). Paracetamol use (and/or misuse) in children in Enugu, South-East, Nigeria. BMC Pediatrics.

[101] Doyon S., Klein-Schwartz W., Lee S., BeuhLer M.C. (2013). Fatalities involving acetaminophen combination products reported to United States poison centers. Clin. Toxicol..

[102] Bronstein A.C., Spyker D.A., Cantilena L.R., Green J., Rumack B.H., Dart R.C. (2011). 2010 Annual Report of the American Association of Poison Control Centers’ National Poison Data System (NPDS): 28th Annual Report. Clin. Toxicol..

[103] Lee W.M. (2017). Public health: acetaminophen (APAP) hepatotoxicity—isn’t it time for APAP to go away?. J. Hepatol..

[104] Mourya A., Mary C., James C., Jose J., Srinivasan R. (2019). A survey on over-the-counter drug usage in the community. J. Drug Deliv. Ther..

[105] Salami K., Adesanwo O. (2015). The practice of self-medication for treatment of illnesses for under-five children by mothers in Ibadan, Nigeria. Res. J. Drug Abuse.

[106] Okunola O.A. (2020). Patterns of self-medication practices by caregivers to under-five children in South-Western Nigeria. Child Care Pract..

[107] Remsing J., Mein S. (2002). Rectal and parenteral paracetamol in combination with NSAIDs for post operative anaesthasia. Br. J. Anaesth..

[108] Heard K., Bui A., Mlynarcheck S.L., Green J.L., Bond G.R., Clark R.F. (2014). Repeated doses of acetaminophen in children: assessment of casuality and dose in reported cases. Am. J. Ther..

[109] Bhutta Z.A., Ahmed T., Black R.E. (2008). What works? Interventions for maternal and child undernutrition and survival. Lancet.

[110] Black R.E., Allen L.H., Bhutta Z.A. (2008). Child undernutrition study group. maternal and child undernutrition: global and regional exposures and health consequences. Lancet.

[111] Alamu E.O., Eyinla T.E., Sanusi R.A., Maziya-Dixon B. (2020). Double burden of malnutrition: evidence from a selected Nigerian population. J. Nutr. Metab..

[112] Bolesta S., Haber S.L. (2002). Hepatotoxicity associated with chronic acetaminophen administration in patients without risk factors. Ann. Pharmacother..

[113] Whitcomb D.C., Block G.D. (1994). Association of acetaminophen hepatotoxicity with fasting and ethanol use. JAMA.

[114] Sabir O.M., Ali A.B., Algemaabi O. (2010). Pattern of liver diseases in Sudanese children. Sudan. J. Med. Sci..

[115] Burki M.K., Orakzai S.A. (2001). The prevalence and pattern of liver disease in infants and children in Hazara Division. J. Ayub Med. Coll. Abbottabad.

[116] Alam M.J., Ahmed F., Mobarak R., Arefin S., Tayab A., Tahera A., Mahmud S. (2010). Pattern of liver diseases in children admitted in Dhaka Shishu Hospital. Int. J. Hepatol..

[117] Rumack B.H. (2004). Acetaminophen misconceptions. Hepatology.

[118] Vitols S. (2003). Paracetamol hepatotoxicity at therapeutic doses. J. Intern. Med..

[119] You M.A., Nam S.M., Son Y.J. (2015). Parental experiences of medication administration to children at home and understanding of adverse drug events. J. Nurs. Res..

[120] Zimmerman H.J., Maddrey W.C. (1995). Acetaminophen (paracetamol) hepatotoxicity with regular intake of alcohol: analysis of instances of therapeutic misadventure. Hepatology.

[121] Draganov P., Durrence H., Cox C., Reuben A. (2000). Alcohol-acetaminophen syndrome. Postgrad. Med..

[122] Vale J.A., Proudfoot A.T. (1995). Paracetamol (acetaminophen) poisoning. Lancet.

[123] Riordan S.M., Williams R. (2002). Alcohol exposure and paracetamol-induced hepatotoxicity. Addict. Biol..

[124] Tanaka E., Yamazaki K., Misawa S. (2000). Update: the clinical importance of acetaminophen hepatotoxicity in non-alcoholic and alcoholic subjects. J. Clin. Pharm. Ther..

[125] Comporti M., Signorini C., Leoncini S., Gardi C., Ciccoli L., Giardini A., Vecchio D., Arezzini B. (2010). Ethanol-induced oxidative stress: basic knowledge. Genes Nutr..

[126] Dart R.C., Kuffner E.K., Rumack B.H. (2000). Treatment of pain or fever with paracetamol (acetaminophen) in the alcoholic patient: a systematic review. Am. J. Ther..

[127] Kuffner E.K., Dart R.C., Bogdan G.M., Hill R.E., Casper E., Darton L. (2001). Effect of maximal daily doses of acetaminophen on the liver of alcoholic patients: a randomized, double-blind, placebo-controlled trial. Arch. Intern. Med..

[128] Lauterburg B.H. (2002). Analgesics and glutathione. Am. J. Ther..

[129] Makin A., Williams R. (2000). Paracetamol hepatotoxicity and alcohol consumption in deliberate and accidental overdose. QJM.

[130] Rotundo L., Pyrsopoulos N. (2020). Liver injury induced by paracetamol and challenges associated with intentional and unintentional use. World J. Hepatol..

[131] Michna E., Duh M.S., Korves C., Dahl J.L. (2010). Removal of opioid/acetaminophen combination prescription pain medications: assessing the evidence for hepatotoxicity and consequences of removal of these medications. Pain Med..

[132] Bunchorntavakul C., Reddy K.R. (2013). Acetaminophen-related hepatotoxicity. Clin. Liver Dis..

[133] Wilkes J.M., Clark L.E., Herrera J.L. (2005). Acetaminophen overdose in pregnancy. South Med. J..

